# Association between *PIK3CA* activating mutations and outcomes in early-stage invasive lobular breast carcinoma treated with adjuvant systemic therapy

**DOI:** 10.2478/raon-2023-0027

**Published:** 2023-06-21

**Authors:** Domen Ribnikar, Valentina Jeric Horvat, Ivica Ratosa, Zachary W Veitch, Biljana Grcar Kuzmanov, Srdjan Novakovic, Erik Langerholc, Eitan Amir, Bostjan Seruga

**Affiliations:** Department of Medical Oncology, Institute of Oncology Ljubljana, Ljubljana, Slovenia; Faculty of Medicine Ljubljana, University of Ljubljana, Ljubljana, Slovenia; Department of Radiation Oncology, Institute of Oncology Ljubljana, Ljubljana, Slovenia; Division of Medical Oncology and Hematology, Royal Victoria Hospital, Bariie, Ontario, USA; Department of Pathology, Institute of Oncology Ljubljana, Ljubljana, Slovenia; Department of Molecular Diagnostics, Institute of Oncology Ljubljana, Ljubljana, Slovenia; Institute of Biostatistics and informatics, Faculty of Medicine Ljubljana, University of Ljubljana, Ljubljana, Slovenia; Division of Medical Oncology and Hematology, University of Toronto and Princess Margaret Cancer Centre, Toronto, Canada

**Keywords:** invasive lobular carcinoma, PIK3CA mutation, endocrine therapy, genomics of invasive lobular carcinoma

## Abstract

**Background:**

The aim of the study was to evaluate the independent prognostic role of *PIK3CA* activating mutations and an association between *PIK3CA* activating mutations and efficacy of adjuvant endocrine therapy (ET) in patients with operable invasive lobular carcinoma (ILC).

**Patients and methods:**

A single institution study of patients with early-stage ILC treated between 2003 and 2008 was performed. Clinicopathological parameters, systemic therapy exposure and outcomes (distant metastasis-free survival [DMFS] and overall survival [OS]) were collected based on presence or absence of PIK3CA activating mutation in the primary tumor determined using a quantitative polymerase chain reaction (PCR)-based assay. An association between PIK3CA mutation status and prognosis in all patient cohort was analyzed by Kaplan-Meier survival analysis, whereas an association between PIK3CA mutation and ET was analyzed in estrogen receptors (ER) and/or progesterone receptors (PR)-positive group of our patients by the Cox proportional hazards model.

**Results:**

Median age at diagnosis of all patients was 62.8 years and median follow-up time was 10.8 years. Among 365 patients, PIK3CA activating mutations were identified in 45%. PIK3CA activating mutations were not associated with differential DMFS and OS (p = 0.36 and p = 0.42, respectively). In patients with PIK3CA mutation each year of tamoxifen (TAM) or aromatase inhibitor (AI) decreased the risk of death by 27% and 21% in comparison to no ET, respectively. The type and duration of ET did not have significant impact on DMFS, however longer duration of ET had a favourable impact on OS.

**Conclusions:**

PIK3CA activating mutations are not associated with an impact on DMFS and OS in early-stage ILC. Patients with PIK3CA mutation had a statistically significantly decreased risk of death irrespective of whether they received TAM or an AI.

## Introduction

Breast cancer represents a spectrum of heterogeneous diseases with different clinical behaviour and response to specific systemic therapy. Invasive lobular carcinoma (ILC) is the second most frequent subtype of breast cancer, representing around 10% of all breast cancer cases.^1^

It has been demonstrated that ILC is a special disease entity that differs from the more common invasive breast carcinomas such as invasive ductal carcinoma (IDC). These differences include risk factors, histological and clinical characteristics, transcriptional signatures and genomic profiles.^2,3,4^ ILCs usually arise in postmenopausal women, are larger in size at the time of diagnosis due to insidious nature of its growth, but are typically of lower histological grade. The majority of ILC tumors express hormone receptors such as estrogen receptors (ER), progesterone receptors (PR) but they less commonly exhibit human epidermal growth factor receptor-2 (HER-2) overexpression or amplification.^5^ Data about prognosis of ILC vary substantially; studies have demonstrated better^6,7^, the same^8^, and worse long-term overall survival (OS) in comparison to unselected invasive breast cancers.^9^

Additionally, it has been reported that ILC is less responsive to chemotherapy (ChT).^10,11,12^ However, these tumors respond well to endocrine therapy (ET) given their biological profile.^13^ Results of an important retrospective study clearly showed an OS benefit of adjuvant ET for patients with ILC.^14^

Desmedt *et al.* conducted the largest genomic study of ILC and found that mutations in *PIK3CA* (phosphatidylinositol, catalytic unit), *HER* and *ESR1* (estrogen receptor 1) genes are more frequently present in ILC tumors that in other invasive breast cancers. Furthermore, in their study, approximately 50% of ILC tumors harboured PIK3CA-PTEN-AKT1 signaling alterations. Of note, *PIK3CA* mutation was associated with lower histological grade and lower Ki-67 (proliferating) index.^15^ Mutations of *PIK3CA* gene are the most common somatic genetic alterations in ER-positive breast cancer and are associated with favorable breast cancer characteristics such as smaller tumor size, lower grade, ER positivity and increasing age.^16^

Investigators of the BIG 1–98 study which compared letrozole monotherapy and letrozole switching strategy to tamoxifen (TAM) monotherapy reported that postmenopausal patient population with ILC may derive greater benefit from letrozole (AIs) than patients with IBC, NOS (invasive breast carcinoma, no otherwise specified).^17^ Furthermore, additional post-hoc analysis of the BIG 1–98 study showed that irrespective of the histological subtype of breast cancer tumors with *PIK3CA* mutation derive greater benefit from letrozole than from tamoxifen.^18^ There are no data about the exact number of ILC patients with present *PIK3CA* mutation in the BIG 1–98 study; however in the large meta-analysis by Zardavas *et al.* there were 366 out of 951 (39%) patients who had *PIK3CA* mutated ILC.^16^

In this study, we aimed to evaluate the independent prognostic role of *PIK3CA* activating mutations and an association between *PIK3CA* activating mutations and efficacy of ET in patients with operable ILC. We hypothesized that the presence of *PIK3CA* activating mutations in primary ILC is associated with longer distant metastasis-free survival and overall survival and greater benefit of AIs and extended ET as compared to standard 5-year treatment with tamoxifen.

## Patients and methods

### Patients

After obtaining approval from the institutional review committee and ethical approval of the Ministry of Health of the Slovenian Republic (#0120-323/2019), we performed a retrospective analysis of a cohort of patients with early-stage ILC identified from a pathology database at the Institute of Oncology Ljubljana. Eligible patients were treated between January 1^st^ 2003 and December 31^st^ 2008, thereby allowing at least 10 years of follow-up at the time of data analysis.

### Clinicopathological characteristics and determination of the *PIK3CA* mutation

Formalin-fixed and paraffin embedded (FFPE) and hematoxylin and eosin stained tumor slides of all included patients were reviewed by an experienced breast pathologist (BGK) to ensure that the diagnosis of ILC and its subtype(s) were correct. We collected the following clinicopathological parameters from each patient's from the existing pathology reports: age at diagnosis, subtype of ILC, tumor size, nodal status, grade, mitotic count, ER and PR expression, HER-2 overexpression or amplification, lymphovascular invasion (LVI) and perineural invasion (PNVI). Ki-67 labeling index and tumor infiltrating lymphocytes (TILs) were additionally determined retrospectively by a single breast cancer pathologist as these biomarkers were not assessed routinely in years of the cohort inception. Tumor grading was classified according to the Bloom-Richardson-Elston classification.^19^ ER and PR expression and HER-2 status were determined using the American Society of Clinical Oncology/College of American Pathologists guidelines.^20,21^ Proliferation index Ki-67 was estimated with DAKO, Glostrup antibody MIB1 according to recommendations from the International Ki-67 in Breast Cancer Working Group.^22^ For the evaluation of TILs the recommendations of the 2014 International TILs Working Group were followed.^23^ *PIK3CA* status (wild-type or mutated) was determined for each patient. DNA isolation from the FFPE tumor samples was performed by macrodissection of tumor tissue from tumor slices containing at least 70% tumor cells. Isolation was performed using the MagMAX FFPE DNA/RNA Ultra Kit (Applied Biosystems, Thermo Fisher Scientific, Austin, Tx, USA) according to the manufacturer's instructions. Isolated DNA was used to identify five common mutations in the *PIK3CA* gene using the PIK3CA Mutation Analysis Kit (EntroGen, Inc. Woodland Hills, CA, USA) according to the manufacturer's protocol. This is a quantitative polymerase chain reaction (PCR) based assay that detects five hot spot mutations in the *PIK3CA* gene (NM_006218.2): c.1624G > A p.(Glu542Lys), c.1633G > A p.(Glu545Lys), c.1633G > C p.(Glu545Gln), c.3140A > G p.(His1047Arg) and c.3140A > T p.(His1047Leu). These five hot spot mutations represent around 80% of all *PIK3CA* mutations.^24^

### Systemic treatment

We collected all data about systemic therapy for each patient included into the study such as whether the patient received ChT, ET and HER-2-targeted therapy. We also collected data about the type of ChT (cyclophosphamide – metotrexate-5-fluorouracil regimen (CMF), anthracycline-based chemotherapy, anthracyclines and taxanes, taxanes without anthracyclines), type of ET (tamoxifen monotherapy, AI monotherapy or a switching approach [TAM-AI]) and duration of ET (up to 5 years or more than 5 years).

### Statistical analysis

The outcomes of interest were distant metastasis-free survival (DMFS) and overall survival (OS) defined as time from diagnosis (date of surgery) to distant relapse or death (whichever occurred first) and as time from diagnosis to death from any cause, respectively. The last date of a follow-up was October 31^st^ 2021. Data were analyzed using R version 4.1.2 (Vienna, Austria). Descriptive statistics were used to describe patient characteristics. Age and duration of therapy were measured in years, tumor size was measured in millimeters, Ki-67 index was measured as a proportion of positive tumor cells and logarithmised and for the number of positive axillary lymph nodes the square root was taken of in order to normalize the distribution of the variables and improve model fit. DMFS and OS were estimated using Kaplan-Meier analysis. Log-rank tests were performed to compare the survival of *PIK3CA* mutated and *PIK3CA* non-mutated cohorts of patients. Four Cox proportional hazard models were used to assess the impact of ET on survival of ER and/or PR-positive *PIK3CA* mutated patients: a simple and an advanced model for each of DMFS and OS. The simple Cox model regressed on only three variables; age at diagnosis, prior duration of tamoxifen and prior duration of AI therapy. The advanced model regressed on additional variables which are considered known or possible prognostic factors: tumor size, nodal status, histological grade, Ki-67 index, PR expression and location of *PIK3CA* mutation (exon 9 or exon 20), respectively. To avoid immortal time bias, any prior duration of tamoxifen and any prior duration of AI therapy were considered as time-dependent variables, while all other variables were known at a patients' entry in the study (at the time of surgery). Finally, adjustment for multiple significance testing was performed using Holm's method. An adjusted p-value ≤ 0.05 was considered statistically significant.

## Results

### Patients and their tumors

**FIGURE 1.**
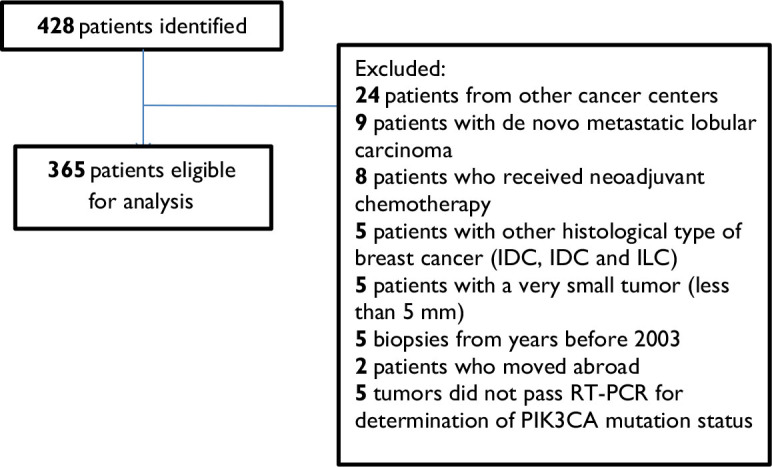
Consort diagram.

Among an initial cohort of 428 patients, we excluded 24 patients who were treated in other cancer centers in Slovenia, nine patients who had de novo metastatic lobular cancer, eight patients who received neoadjuvant ChT, five patients with other histological subtypes of breast cancer (IBC, NOS, ILC and IBC, NOS), five patients with very small tumors (less than 5 mm in size) and five patients whose biopsies were from years before 2003. Two patients were lost very early in the follow up as they moved out of country, so they were excluded from the analysis. *PIK3CA* mutation status could not be determined in five cases due to technical issues with the samples. This resulted in an analytic cohort of 365 patients (Figure 1; Consort diagram, for patient selection schema). Characteristics of included patients and their tumors are presented in Table 1. Among all patients, 164 (45%) patients had *PIK3CA* mutated ILC. Of these 78 (48%) patients had *PIK3CA* mutations in the helical domain (exon 9) and 76 (46%) patients had the mutation present in the kinase domain (exon 20). Five patients (1%) had dual *PIK3CA* mutations on exon 9 and five patients (1%) had *PIK3CA* mutations on both, exon 9 and 20. As shown in Table 1, *PIK3CA* mutated and *PIK3CA* non-mutated cohort of patients were well balanced according to prognostic tumor characteristics.

**TABLE 1.** Characteristics of the included patients and their tumors

**Characteristic**	**All patients (n = 365; 100%)**	***PIK3CA* mutated (n = 164; 45%)**	***PIK3CA* non-mutated (n = 201; 55%)**
**Median age (range) (yrs)**	62.8 (33–90)	63.1	62.4
**Median tumor size (mm)**	21	19	21
**Nodal status**			
**N0**	209 (57)	100 (61)	109 (54)
**N1**	84 (23)	37 (22)	47 (23)
**N2**	26 (7)	1 (7)	15 (8)
**N3**	46 (13)	16 (10)	30 (15)
**Tumor grade**			
**G1**	52 (14)	20 (12)	32 (16)
**G2**	270 (74)	134 (82)	136 (68)
**G3**	43 (12)	10 (6)	33 (16)
IHC^*^ subtype			
**ER+/PR+/HER2−**	299 (82)	136 (46)	159 (54)
**ER+/PR−/HER2−**	39 (11)	14 (36)	25 (64)
**HER2 +**	20 (6)	9 (45)	11 (55)
**ER−/PR−/HER2−**	7 (2)	2 (29)	5 (71)
**Median Ki-67 (range) (%)**	3 (1–50)	2.5 (1–50)	3 (1**–**40)
**Mitotic score**			
**M1**	284 (78)	138 (84)	146 (73)
**M2**	55 (15)	19 (12)	36 (18)
**M3**	26 (7)	7 (4)	19 (9)
**Presence of LVI**	24 (7)	4 (2)	20 (10)
**Median TILs (range) (%)**	3 (1**–**50)	3	3
**Median follow-up time (range) (yrs)**	10.8 (0.1**–**18.6)	10.8	10.7

ER = estrogen receptor; HER = 2–human epidermal growth factor receptor-2;

* IHC = immunohistochemically defined subtype; PR = progesterone receptor; LVI = lymphovascular invasion; TILs = tumor-infiltrating lymphocytes

78 patients had PIK3CA mutation on exon 9, 76 patients on exon 20; five patients had dual PIK3CA mutation on exon 9 and five patients had both, exon 9 and exon 20 PIK3CA mutations

Systemic therapy exposure of all included patients is shown in Table 2. As expected, the majority of patients received ET with either TAM (106, 29%), AIs (127, 35%) or a switch approach TAM-AI (107, 29%). Ninety-one (25%) patients received ChT and ET and only 13 (4%) patients received ChT alone. Among 25 patients who did not receive any ET, 10 patients were *PIK3CA* mutated. ET agents were well balanced between *PIK3CA* mutated and *PIK3CA* non-mutated patients as shown in Table 2. Median duration of ET was 5 years in both groups of patients. 191 (52%) patients had extended ET and the longest duration of extended ET was 16.7 years.

**TABLE 2.** Distribution of systemic therapy in all patients and according to PIK3CA mutation status

**Systemic therapy**	**All pts** **N (%)**	***PIK3CA* mutated pts** **N (%)**	***PIK3CA* non-mutated pts** **N (%)**
**None**	12 (3)	5 (3)	7 (3)
**ET only**	249 (68)	115 (70)	134 (67)
**ChT only**	13 (4)	5 (3)	8 (4)
**ET and ChT**	91 (25)	39 (24)	52 (26)
**None ET**	25 (7)	10 (6)	15 (7)
**ET with TAM**	106 (29)	45 (27)	61 (30)
**ET with AIs**	127 (35)	54 (33)	73 (37)
**Sequence TAM-AI**	107 (29)	55 (34)	52 (26)
**Median duration (range) of ET**	5 (0.2–16.7)	5 (0.4–16.7)	5 (0.2–11.2)

AIs = aromatase inhibitors; ChT = chemotherapy; ET = endocrine therapy; pts = patients, TAM = tamoxifen (TAM)

### An association between *PIK3CA* mutation status and disease outcome

**FIGURE 2.**
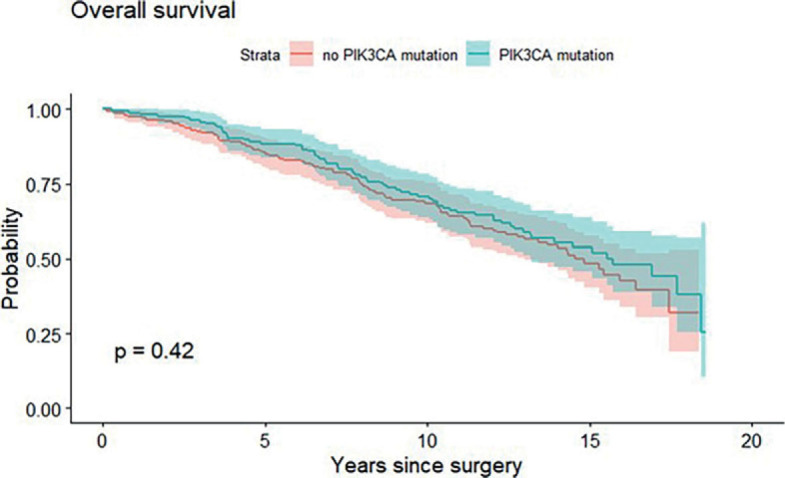
Kaplan-Meier graph showing association between PIK3CA mutation and overall survival.

**FIGURE 3.**
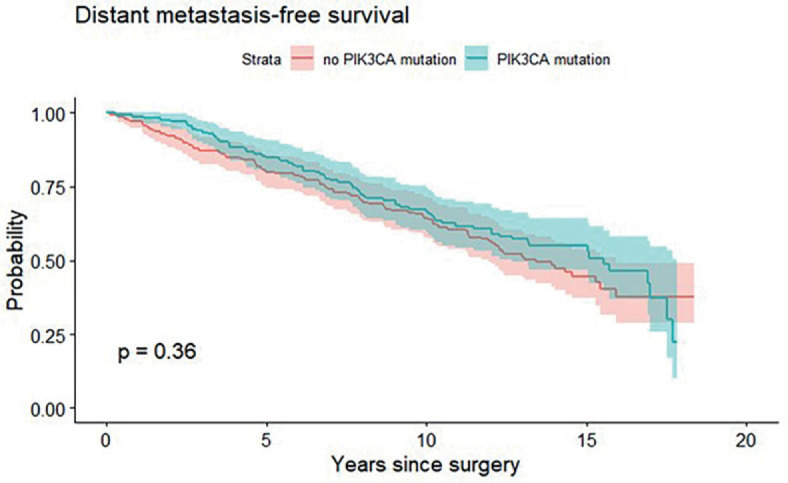
Kaplan-Meier graph showing association between PIK3CA mutation and distant metastasis-free survival

Median OS was 15.7 years for patients with *PIK3CA* mutation and 14.6 years for patients without *PIK3CA* mutation. Furthermore, median DMFS for *PIK3CA* mutated and for *PIK3CA* non-mutated ILC patients was 15.6 and 13.4 years, respectively. The presence of *PIK3CA* mutation in early-stage ILC patients was not associated with differential OS (p = 0.42, Figure 2) or DMFS (p = 0.36, Figure 3). Overall, 81 (22%) patients developed distant metastases, of these 48 (24%) were *PIK3CA* non-mutated and 33 (16%) were *PIK3CA* mutated. Twenty (26%) patients of those who developed distant metastases had *PIK3CA* mutation on exon 20, 12 (15%) had *PIK3CA* mutation on exon 9 and only one (5%) patient had both, exon 9 and 20 *PIK3CA* mutation.

### An association between type and duration of ET and disease outcome in patients with *PIK3CA* mutation

#### The effect of the type of systemic ET and its duration on DMFS

Patients who received AIs in comparison to those who received tamoxifen were on average 10 years older at the time of diagnosis, therefore a Cox proportional hazards model was used for adjustment. In a simple model, neither type of ET nor its duration and patient age at the time of diagnosis were associated with the risk of relapse (Table 3). In advanced Cox proportional hazards model only a higher number of positive axillary lymph nodes increased the risk of distant relapse (HR 1.64, adj.p < 0.001; see Table 4).

**TABLE 3.** Simple Cox proportional hazards model for DMFS in the PIK3CA mutated patient cohort

**Characteristic**	**HR**	**95% CI**	**Adj p**
**Age**	0.99	0.96–1.02	1.0
**Duration of TAM**	0.92	0.75–1.14	1.0
**Duration of AIs**	1.07	0.90–1.28	1.0

Adj p = adjusted p value; AIs = aromatase inhibitors; CI = confidence interval, HR = hazard ratio; TAM = tamoxifen,

**TABLE 4.** Advanced Cox proportional hazards model for DMFS in the PIK3CA mutated patient cohort

**Characteristic**	**HR**	**95% CI**	**Adj p**
**Age**	1.01	0.97–1.05	1.0
**Tumor size**	0.99	0.57–1.71	1.0
**Grade**			
**G1**	Ref		
**G2**	0.91	0.26–3.22	1.0
**G3**	1.03	0.16–6.83	1.0
**Ki-67**	1.35	0.93–1.95	1.0
**PR**	1	0.99–1.02	1.0
***PIK3CA* status**			
**Other than exon 20**	Ref		
**Only exon 20**	2.19	1.01–4.80	0.582
**No. of positive axillary LNs**	1.64	1.30–2.06	< 0.001
**Duration of TAM**	1.02	0.81–1.29	1.0
**Duration of AIs**	1.06	0.87–1.28	1.0

Adj p = adjusted p value; AIs = aromatase inhibitors; CI = confidence interval; HR = hazard ratio; LNs = lymph nodes; No. = number; PR = progesterone receptor; TAM = tamoxifen

#### The effect of the type of systemic ET and its duration on OS

We found that each year of aging increased the risk of death by 5%. Each year of treatment with tamoxifen decreased the risk of death by 27% in comparison to no ET for a patient of the same age (Table 5). Similarly, each year of treatment with AIs decreased the risk of death by 21% compared to no ET for a patient of the same age (Table 5).

**TABLE 5.** Simple Cox proportional hazards model for OS in the PIK3CA mutated patient cohort

**Characteristic**	**HR**	**95% CI**	**Adj p**
**Age**	1.05	1.02–1.07	< 0.001
**Duration of TAM**	0.73	0.61–0.88	0.002
**Duration of AIs**	0.79	0.67–0.93	0.005

Adj p = adjusted p value; AIs = aromatase inhibitors; CI = confidence interval; HR = hazard ratio; TAM = tamoxifen

In the advanced Cox proportional hazards model age at the time of diagnosis, grade 3 and higher number of positive axillary lymph nodes were all associated with higher risk of death (HR 1.05, *adj.* p = 0.014, HR 5.5, *adj.* p = 0.040 and HR 1.58, *adj.* p < 0.001, respectively). We also found that longer duration of ET with either tamoxifen or an AI had a favorable impact on OS (p = 0.010 for tamoxifen and p = 0.010 for an AI, respectively). In a time-dependent Cox analysis each year of prior treatment with tamoxifen decreased the risk of death by 32% in comparison to no prior ET for a patient with the same characteristics. Similarly, to tamoxifen, each year of prior AI therapy decreased the risk of death by 27% compared to no prior ET for a patient with the same characteristics (Table 6).

**TABLE 6.** Advanced Cox proportional hazards model for OS in the PIK3CA mutated patient cohort

**Characteristic**	**HR**	**95% CI**	**Adj p**
**Age**	1.05	1.02–1.08	0.014
**Tumor size**	1.38	0.90–2.10	0.84
**Grade**			
**G1**	Ref		
**G2**	1.41	0.59–3.38	1.0
**G3**	5.52	1.67–18.18	0.04
**Ki-67**	0.94	0.72–1.22	1.0
**PR**	1	0.99–1.01	1.0
***PIK3CA* status**			
**Other than exon 20**	Ref		
**Only exon 20**	1.32	0.79–2.21	1.0
**No. of positive axillary LNs**	1.58	1.32–1.88	< 0.001
**Duration of TAM**	0.68	0.55–0.86	0.01
**Duration of AI**	0.73	0.60–0.88	0.01

Adj p = adjusted p value; AIs = aromatase inhibitors; CI = confidence interval; HR = hazard ratio; No. = number; PR = progesterone receptor; TAM = tamoxifen

## Discussion

ILC of the breast is a distinct entity with unique clinical, histological and molecular characteristics that differ from more common invasive breast cancer subtypes. The majority of older studies demonstrated that the outcome of ILC patients was better, however more recent data have suggested ILC has worse outcome compared to invasive ductal carcinoma, especially in the long term. It seems that delay and difficulty in early diagnosis, acquired resistance to conventional therapy and risk of late relapse pose challenges in management of patients with ILC.^25^

In this retrospective study our main goal was to evaluate the association between the *PIK3CA* mutational status and prognosis in patients with operable ILC. We found that *PIK3CA* mutation status has no association with either DMFS or OS. The median OS was 15.7 years for patients with *PIK3CA* mutation and 14.6 years for patients with *PIK3CA* wild type ILC.

*PIK3CA* mutations are the most frequent somatic genetic alterations in ER+/HER-2− breast cancer.^18^ Prior data in early-stage disease have demonstrated a favorable disease outcome in patients harboring *PIK3CA* mutations.^16^ At least to some extent this could be explained by a relative mutual exclusion of other somatic genetic alterations which are associated with higher proliferation and increased risk of distant relapse such as *TP53* mutations and amplifications of *MYC* (MYC proto - oncogene) gene.^18^ Furthermore, recent work has shown that ultra-low risk breast cancers have higher expression scores for the *PIK3CA*-mutation-associated genes.^26^

Previous data exploring the prognostic effect of specific somatic mutations in ER+/HER-2− early-stage breast cancer have shown that *PIK3CA* and *MAP3K1* (mitogen-activated protein kinase 1) mutations co-associate and patients with tumors harboring both mutations have a more favorable clinical course than those with only one gene mutation or without any of these two mutations.^27^ This may suggest that *PIK3CA* mutation alone is not prognostic in general breast cancer patient population, nor is it in ILC specifically.

The majority of first- and second-generation gene-expression profiles that have been used for molecular prognostication in early-stage breast cancer were developed initially in invasive ductal carcinomas and therefore their use in ILC is uncertain. However recent data have shown they could also be informative for prognostication in ILC patients. ILC tumors are quite homogenous clinically especially relating to classical prognostic features. The majority are grade 2, ER and PR positive, HER-2 negative and have a relatively low proliferative activity.^28^ In contrast, it has been shown recently that a 194-gene signature called LobSig may be the best in prognosticating ILC tumors. LobSig outperformed Nottingham Prognostic Index (NPI), Prosigna ROR, Oncotype Dx and Genomic Grade Index (GGI) in a multivariate Cox proportional hazards model, especially in grade 2 ILC moderate NPI cases.^29^ The authors reported that ILCs that were associated with a high-risk score were enriched for mutations in *HER-2, HER-3, TP53* (TP53 tumor suppressor gene), *ROS1* (ROS proto-oncogene 1, receptor tyrosine kinase) and *AKT1* (AKT serin - threonin protein kinase 1) genes. In contrast, those ILCs that had a good outcome (a low-risk score) had relatively few genomic alterations and interestingly, *PIK3CA* mutations were more common in the latter group than in the high risk ILC group. Based on all data discussed above it seems that *PIK3CA* mutation alone may not be an optimal genetic alteration to stratify ILC patients into better and worse prognostic group but rather an integration of several concurrent genetic alterations might outperform *PIK3CA* mutation in an individual tumor.

We also aimed to explore the predictive effect of *PIK3CA* mutation on benefit from ET. Efficacy of AIs as compared to tamoxifen have previously been explored and reported.^18^ In the analysis from the randomized BIG 1–98 trial, the authors demonstrated a greater magnitude of benefit with an AI letrozole in comparison to tamoxifen for patients with *PIK3CA* mutated early-stage breast cancer (HR 0.18; 95% CI 0.06 – 0.50). Additionally, the same study demonstrated that letrozole was associated with a significantly reduced risk of disease - free survival event (DFS) in patients with luminal A and luminal B ILC concluding the magnitude of benefit from AI was greater in patients with ILC versus invasive ductal carcinomas.^30^ In our study we did not find any substantial differences between the effect of tamoxifen and AIs in *PIK3CA* mutated early-stage ILC patients when compared to patients who did not receive any adjuvant therapy. We found that each year of treatment with tamoxifen decreased the risk of death by 27% in comparison to no ET for a patient of the same age. Similarly, each year of AI therapy decreased the risk of death by 21% compared to no ET for a patient of the same age. In addition, in an advanced Cox model we found similar results. The duration of any ET, either with tamoxifen or an AI, was statistically significantly associated with OS. In patients with *PIK3CA* mutation each year of tamoxifen and AIs treatment decreased the risk of death by 32% and 27% in comparison to a patient with the same tumor characteristics who did not receive any ET, respectively. However, we did not find any associations between type and duration of ET and DMFS. Only a higher number of positive axillary lymph nodes increased the risk of distant relapse of ILC in our cohort.

An interesting study evaluating the relationship of *PIK3CA* mutations and response to short-term neoadjuvant AI therapy demonstrated that the presence of *PIK3CA* mutation did not preclude a response to AIs. In this study suppression of Ki-67 proliferating index, which is a validated intermediate endpoint biomarker for endocrine sensitivity was used as a surrogate marker of response.^31^ Furthermore, another study showed that patients with *PIK3CA* mutated early-stage breast cancer either treated with tamoxifen or untreated had improved outcome in comparison to *PIK3CA* non-mutated patients.^32^ These data were concordant with previous findings from in vitro study showing that *PIK3CA* mutated cell lines were more sensitive to tamoxifen compared to *PIK3CA* wild type cell lines.^33^

Our study has several limitations. First, due to the retrospective nature of this study our findings need to be interpreted with caution and possibly validated in the prospective setting. Second, our cohort of ILC patients is very likely molecularly heterogeneous which limits the generalizability of our findings. Third, longer duration of ET in this study is associated with significantly improved OS but not DMFS. This indicates that the impact of immortal time bias may persist despite the use of time-dependent Cox model in our analysis. Fourth, we used a PCR based assay which detects five hot spot mutations in the *PIK3CA* gene representing about 80% of all *PIK3CA* activating mutations. Results could be different if we used an alternative method for determination of all *PIK3CA* mutations such as next generation sequencing (NGS). Finally, the biggest limitation of our study is that despite the use of Cox models confounding by indication for treatment might still have an impact on our results.

In conclusion, the presence of *PIK3CA* mutation was not associated with differential DMFS or OS in patients with operable ILC. Adjuvant ET with either tamoxifen or an AI significantly decreased the risk of death in *PIK3CA* mutated ILC cohort. Furthermore, there was no significant difference in efficacy between the two classes of endocrine agents. Longer duration of ET with either tamoxifen or AIs decreases the risk of death in ILC patients with *PIK3CA* mutation. Future prospective studies based on molecular analyses will hopefully give us more exact answer about optimal endocrine agent for specific subpopulations of patients with early-stage ILC.
